# Long-term results of ablation index guided atrial fibrillation ablation: insights after 5+ years of follow-up from the MPH AF Ablation Registry

**DOI:** 10.3389/fcvm.2023.1332868

**Published:** 2024-01-16

**Authors:** N. Fitzpatrick, S. Herczeg, K. Hong, F. Seaver, L. Rosalejos, U. Boles, G. Jauvert, E. Keelan, J. O’Brien, T. Tahin, J. Galvin, G. Széplaki

**Affiliations:** ^1^ Atrial Fibrillation Institute, Mater Private Hospital, Dublin, Ireland; ^2^ Department of Cardiology, Heart and Vascular Centre of Semmelweis University, Budapest, Hungary; ^3^ Health Sciences Centre, UCD School of Medicine, University College Dublin, Dublin, Ireland; ^4^ Department of Cardiology, Mater Misericordiae University Hospital, Dublin, Ireland; ^5^ Department of Medicine, Royal College of Surgeons in Ireland, Dublin, Ireland

**Keywords:** atrial fibrillation, pulmonary vein isolation (PVI), catheter ablation, ablation index, long-term follow-up

## Abstract

**Background:**

Catheter ablation (CA) for symptomatic atrial fibrillation (AF) offers the best outcomes for patients. Despite the benefits of CA, a significant proportion of patients suffer a recurrence; hence, there is scope to potentially improve outcomes through technical innovations such as ablation index (AI) guidance during AF ablation. We present real-world 5-year follow-up data of AI-guided pulmonary vein isolation.

**Methods:**

We retrospectively followed 123 consecutive patients who underwent AI-guided CA shortly after its introduction to routine practice. Data were collected from the MPH AF Ablation Registry with the approval of the institutional research board.

**Results:**

Our patient cohort was older, with higher BMI, greater CHA2DS2-VASc scores, and larger left atrial sizes compared to similar previously published cohorts, while gender balance and other characteristics were similar. The probability of freedom from atrial arrhythmia with repeat procedures is as follows: year 1: 0.95, year 2: 0.92, year 3: 0.85, year 4: 0.79, and year 5: 0.72. Age >75 years (*p* = 0.02, HR: 2.7, CI: 1.14–6.7), BMI >35 kg/m^2^ (*p* = 0.0009, HR: 4.6, CI: 1.8–11.4), and left atrial width as measured on CT in the upper quartile (*p* = 0.04, HR: 2.5, CI: 1–5.7) were statistically significant independent predictors of recurrent AF.

**Conclusion:**

AI-guided CA is an effective treatment for AF, with 95.8% of patients remaining free from atrial arrhythmia at 1 year and 72.3% at 5 years, allowing for repeat procedures. It is safe with a low major complication rate of 1.25%. Age >75 years, BMI >35 kg/m^2^, and markedly enlarged atria were associated with higher recurrence rates.

## Graphical Abstract

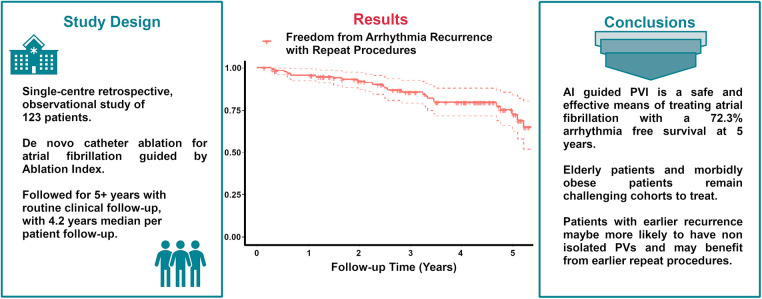

## Introduction

Pulmonary vein isolation (PVI) remains the cornerstone of treatment for atrial fibrillation (AF), particularly in patients who are refractory or intolerant to anti-arrhythmic medications (1). In paroxysmal AF, circumferential lesions around the pulmonary veins (PVs) effectively target source triggers and isolate them from the rest of the left atrium (2). However, PVI for persistent AF remains suboptimal and is associated with higher recurrence rates of AF and atrial tachycardia, often requiring repeat PVI or additional substrate modification (3).

Several targets have been proposed to maximise the durability of ablation lesions, including contact force (CF) and minimum force–time integral (FTI). While the use of CF and FTI metrics has demonstrated improved AF ablation outcomes, up to two-thirds of patients have been reported with at least one reconnected PV at 2–3 months following the initial intervention (4, 5). Therefore, the ablation index (AI) has emerged as a novel strategy for lesion delivery, incorporating power, CF, and time in a weighted formula to achieve enduring PV isolation.

The AI formula provides an accurate measure of ablation lesion quality monitoring as part of the automated lesion tagging software (VisiTag) in the CARTO 3 V4 electroanatomic mapping system (Biosense Webster, Inc., Diamond Bar, CA, USA). Indeed, AI values of 550/400 (anterior/posterior) with an inter-lesion distance (ILD) of ≤6 mm have been associated with higher rates of acute durable PVI isolation and arrhythmia-free survival at 1 year (6, 7). While the use of AI targets in lowering rates of PV reconnection has previously been investigated (7, 8), studies have shown that durable PVI can be achieved with lower AI targets. Lower effective AI targets can be effective (9), specifically with more intensive catheter tip cooling (10), tailoring to left atrial wall thickness (LAWT) (11, 12), and focusing on ostial rather than wide antral lesions (13). Typical follow-up in these reports was limited to 12–18 months. Also, initial studies were limited to patients with paroxysmal (pAF) only without additional lesion sets and therefore did not truly reflect a real-world scenario. We describe, to the best of our knowledge, the first report of 5-year follow-up clinical outcomes using the index-guided catheter ablation strategy for AF.

## Methods

### Study design

We retrospectively enrolled 123 consecutive patients who attended our centre after December 2016 (corresponding with the introduction of AI-guided PVI) and underwent a *de novo* pulmonary vein isolation procedure. Our target was to enrol at least 100 patients who would have long-term regular follow-ups at our institute and a minimum of at least 90 days. Exclusion criteria were kept to a minimum—patients with prior cardiac surgery, patients with prior PVI or left atrial ablation, and any patient not expected to be followed up routinely at our centre were excluded. The patient cohort included both paroxysmal and persistent atrial fibrillation patients. Patients underwent PVI and “PVI plus” procedures as deemed appropriate by the operator based on their subtype of atrial fibrillation, a stepwise ablation strategy, and the degree of low-voltage areas found on mapping of the left atrium; in the cases of the documented atrial flutter, applicable lesions/lines were considered. Institutional review board ethics approval was sought and attained—MMUH/MPH IRB Ethics Approval Reference 1/378/2283.

### Patient follow-up

Patients were kept overnight and routinely discharged on day 1 post-procedure. Patients were reviewed at 6 weeks and again at 3 months with 24-h Holter monitoring prior to their clinic visit, along with resting ECGs on the day of their appointment. They were also provided contact details for office hours and out-of-office hours to report any troublesome symptoms. If patients reported symptoms but were found to be in sinus rhythm on resting ECG, a routine 24-h Holter monitoring was arranged. In some cases, patients documented their arrhythmia with their consumer-grade ECG devices (smartphones/smart ECG devices). If patients remained symptom-free, they were followed up routinely on an annual basis. Patients with implantable cardiac devices had device checks at 6 weeks and at 3 months to determine the AF burden. An exhaustive search of all patient correspondence, written notes, investigation reports (including ECGs, Holters, device checks, echocardiography) and procedural reports (coronary angiography, cardioversions, repeat ablation) was completed to look for recurrence of atrial fibrillation or any atrial arrhythmia. Recurrence was defined as greater than 30 s of documented atrial arrhythmia after a 90-day blanking period was applied. Survival time was calculated until the last patient clinic contact if no recurrence occurred. If a patient had a recurrence, but the exact date was unclear, it was assumed to be 1 day after the last documented date when the patient was arrhythmia-free. Where patients had multiple procedures, survival time was the accumulated arrhythmia-free survival for all their procedures.

### Procedure details

All patients underwent pre-procedure computed tomography of their left atrium (CTLA) to delineate anatomy. All procedures were performed under general anaesthesia. Transoesophageal echocardiography was utilised to guide transseptal puncture and exclude left atrial thrombus. Vascular ultrasound was used to introduce a 7-Fr sheath, an SL0 sheath (Swartz 63 cm SL0 Transseptal Guiding Introducer Sheath; Abbott Laboratories, Chicago, IL, USA) and a steerable sheath (Agilis NXT Steerable Introducer; Abbott Laboratories, Chicago, IL, USA) into the right femoral vein.

Our anticoagulation strategy was as follows: patients on warfarin were continued without interruption, patients on direct oral anticoagulants (DOACs) had their dose held on the day of the procedure, and DOACs were recommenced after a post-procedure echocardiogram to exclude effusion at 3 h post-procedure. Periprocedural anticoagulation was as follows: 125 units of unfractionated heparin per kg after femoral puncture with a target activated clotting time (ACT) of 300–350 s. Heparin infusions of 1,000 units/h were administered via two long sheaths. The heparin bolus was given after venous access and before access to the left atrium.

A deflectable decapolar catheter (14) (Dynamic Deca; Boston Scientific, Natick, MA, United States) is positioned in the coronary sinus (CS) under fluoroscopy guidance. The transseptal puncture was performed using a 71-cm BRK-1 XS needle (Abbott Laboratories, Chicago, IL, USA) via the SL0 sheath. Whenever possible, the first puncture was double-wired. If the patient was in atrial fibrillation, they were cardioverted (200 J synchronised) after a transseptal puncture. Prior to ablation, a 3D map of the left atrium was created with proximal CS pacing at a cycle length of 600 ms with both voltage and activation data using a Lasso circular or PentaRay multielectrode mapping catheter and a CARTO (15) electro-anatomical mapping system (Biosense Webster, Irvine, CA, USA).

Ablation was performed with a SmartTouch Surround Flow DF catheter (16) (Biosense Webster, Irvine, CA, USA) and guided by the ablation index (9) with targets of 370 and a power of 35 W on the posterior and inferior regions while aiming for 480 with a power of 40 W on the anterior and superior regions. Our ablation lesion strategy was as follows: If the patient's initial rhythm was, or if after cardioversion they maintained, sinus rhythm, PVI only was performed using bilateral wide antral circumferential ablation (WACA) (17, 18) lines. If a short distance was left between the bilateral WACA lines on the roof (<1.5 cm), a roof line was completed. If they failed to maintain sinus rhythm, a posterior wall isolation box was performed. If the patient failed to maintain sinus rhythm at this point, an anterior mitral isthmus line was also created. If they failed to maintain sinus rhythm acutely at this point, additional scar homogenisation and superior vena cava (SVC) isolation were performed. If the patient developed an atrial tachycardia at any point, it was mapped and, where possible, ablated. Cavotricuspid isthmus (CTI) ablation was performed in cases of documented typical atrial flutter.

Validation was performed by remapping the left atrium after a 20-min waiting period, ensuring both entry and exit blocks into all pulmonary veins. Where linear ablation lesions were created, the bidirectional block was confirmed across these lines using appropriate differential pacing. Adenosine was not routinely used to test for pulmonary vein isolation.

### Statistical analysis

The normality of data distribution was tested with the Shapiro–Wilk test. Continuous variables were expressed as mean ± standard deviation if normally distributed and medians with interquartile range if non-normally distributed, and dichotomous variables were expressed as percentages. The Student *t*-test or the Mann–Whitney *U*-test was used for unpaired group comparison. Categorical variables were compared by the *χ*^2^ or Fisher exact test and were presented as frequency and percentage. The Kaplan–Meier estimate was used to compare freedom from atrial arrhythmia between subgroups and dependence on different patient and procedural characteristics. The log-rank test and Cox proportional hazards were used to check for statistically significant differences. Multivariable logistic regression was used to assess the association between the different parameters and recurrence rates. Variables were included in the multivariate analysis if their univariate *p*-value was less than 0.1 and they were not collinear (denoted by “C” in the *p*-value column—see Table 1) with an already included variable. *p*-values <0.05 were considered statistically significant. All statistical analyses were performed using R version 4.1.2 (19). Survival curves were truncated to 5.5 years to ensure that at least 10% of the original cohort was still at risk, as it is considered standard in reporting of Kaplan–Meier survival curves.

**Table 1:** Results of the Cox proportional hazard model of univariate and multivariate regression analyses.

	Univariate analysis				Multivariate analysis			
	HR	CI	*p*-value		HR	CI	*p*-value	
Age (years)	1	0.97–1.1	0.48					
<65	1	0.46–2.3	0.95					
≥75	**2** **.** **2**	**0** **.** **94–5**	**0**.**069**	*	2.7	1.14–6.7	0.02	**
Persistent AF	1.2	0.58–2.6	0.59					
CHADSVASs	**1**.**3**	**1–1** **.** **6**	**0**.**029**	**			C	
CCF	1.4	0.5–4.2	0.5					
Hypertension	1.3	0.58–3.1	0.5					
Diabetes	**2**.**6**	**1** **.** **1–6.1**	**0**.**032**	**	1	0.4–2.7	0.97	
Vascular disease	1.2	0.49–2.8	0.73					
Stroke or TIA	1.7	0.51–5.8	0.38					
Female gender	0.61	0.27–1.4	0.23					
BMI	**1**.**1**	**1–1** **.** **2**	**0**.**04**	**			C	
BSA	2.9	0.34–24	0.33					
Normal BMI	1	0.31–3.5	0.94					
Overweight	0.67	0.32–1.4	0.31					
Obese	0.37	0.088–1.6	0.18					
BMI ≥35 kg/m^2^	**4**.**2**	**1** **.** **8–9.7**	**0**.**0009**	**	4.6	1.8–11.4	0.0009	**
Recurrence in the blanking period	**1**.**9**	**0** **.** **89–4**	**0**.**1**	*	1.8	0.8–4.1	0.32	
LA AP	1	0.96–1.1	0.55					
LA width	**1**	**1–1** **.** **1**	**0**.**08**	*			C	
LA 3Q width	**3**	**1** **.** **4–6.5**	**0**.**0046**	**	2.4	1–5.7	0.04	**

BSA, body surface area; LA AP, left atrium anteroposterior diameter; LA width, left atrial width measured on CTLA; LA 3Q width, above the third quartile of LA width; CCF, congestive cardiac failure; CT, computed tomography; TIA, transient ischaemic attack.

Variables were included in the multivariate analysis if their univariate *p*-value was less than 0.1 and they were not collinear (denoted by “C” in the *p*-value column) with an already included variable.

Bold denotes univariate *p* value ≤0.1 and hence included in multivariate analysis.

* *p*-values <0.1.

** Statistically significant *p*-values, i.e., <0.05.

## Results

### Patient characteristics

A total of 123 patients were enrolled (mean age: 65.3 **± **9.8 years, 74.8% men). Table 2 details the demographic and baseline data of the study participants. A total of 49% of patients studied had persistent atrial fibrillation. Fifty-four (43%) patients were on class Ic/III anti-arrhythmic drugs (AAD) prior to ablation, and 10 (8%) patients continued their AAD after the blanking period. Seventeen patients exhibited a documented reduced left ventricular systolic function, and six exhibited a normalised left ventricular function after the procedure, where patients responded to appropriate heart failure therapies or systolic dysfunction was tachycardia-mediated.

**Table 2:** Patient characteristics.

Patient characteristics (*n* = 123)	
Male, *n* (%)	92 (74.8)
Age, mean ± SD	65.3 ± 9.8
Age ≥75 years, *n* (%)	23 (18.7)
BMI, kg/m^2^, mean ± SD	28.7 ± 4.5
BMI ≥35 kg/m^2^, *n* (%)	13 (10.6)
Persistent AF, *n* (%)	60 (48.8)
CHADSVASs score, mean ± SD	2.1 ± 1.5
Heart failure, *n* (%)	11 (8.9)
Hypertension, *n* (%)	72 (58.5)
Diabetes, *n* (%)	13 (10.6)
Vascular disease, *n* (%)	26 (21.1)
Stroke or TIA, *n* (%)	7 (5.7)
LA AP. mm, mean ± SD	46.6 ± 7.45
LA width, mm, mean ± SD	72.37 ± 9.6

LA AP, left atrial anteroposterior diameter measured by computed tomography; TIA, transient ischaemic attack; SD, standard deviation.

### Freedom from arrhythmia recurrence

**Figure 1:**
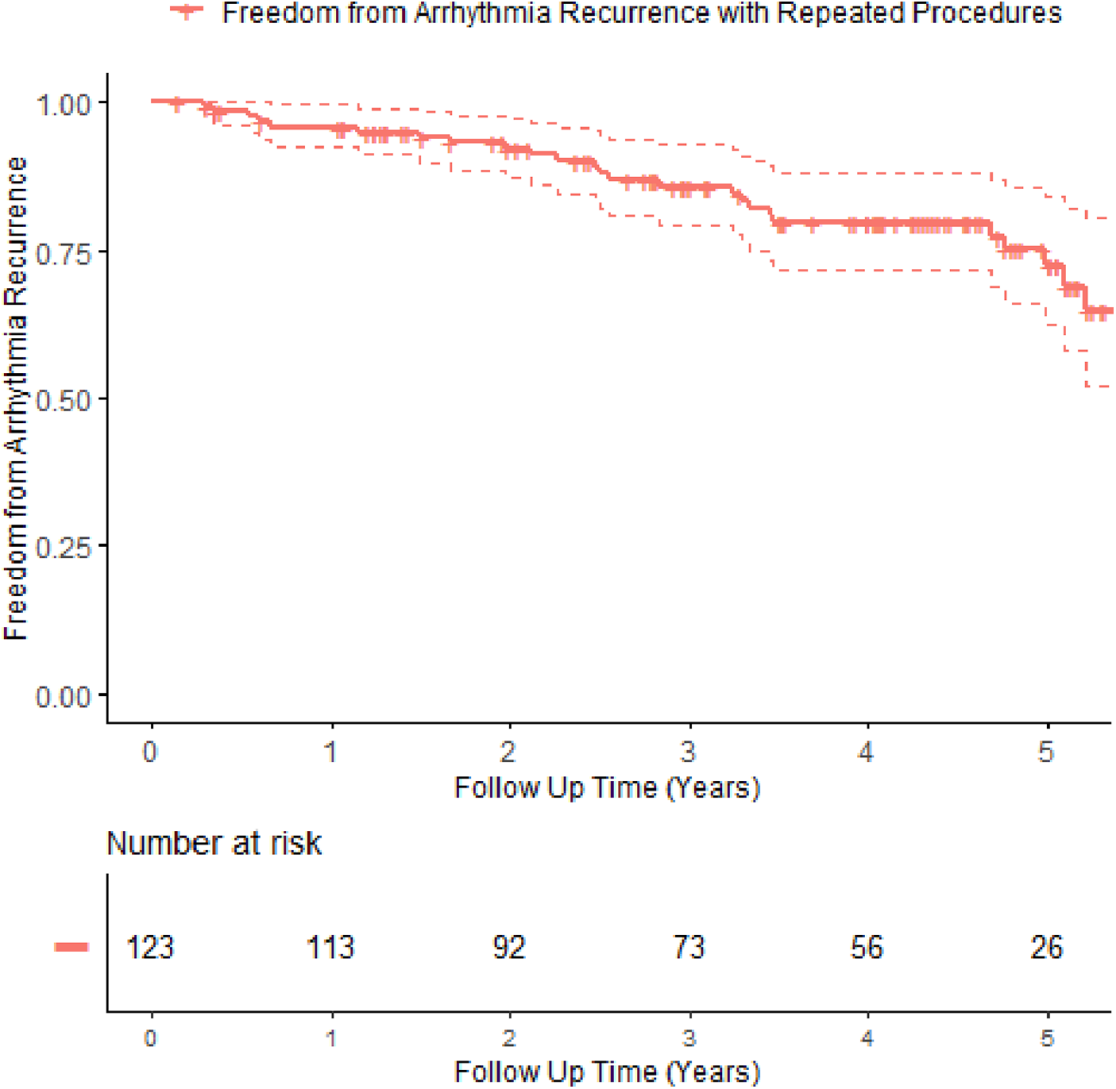
Kaplan–Meier survival curve with 95% confidence interval showing freedom from arrhythmia recurrence with repeat procedures. +denotes censor.

Patients were followed up for up to 6 years after their ablation (median follow-up: 4.2 years, IQR: 2.75, 5.05). Freedom from arrhythmia recurrence, allowing only for the index procedure and repeat procedure, is detailed in Table 3. Allowing for repeat procedures, Figure 1 describes the arrhythmia free survival curve. Median arrhythmia-free survival was 5.6 years.

**Table 3:** Arrhythmia-free survival.

Model/year	1	2	3	4	5
Index procedure only	81.6%	76%	69.8%	63.7%	58.7%
With repeat procedures	95.8%	92%	85.7%	79.5%	72.3%

Out of 123 patients, 44 (35.7%) had a least one recurrence during follow-up. Of these 44 patients, 34 (77% of recurrences) had atrial fibrillation; of these 34 patients, 29% had undergone a PVI “plus” procedure, while 10 patients (40% PVI “plus”) experienced atypical atrial flutter.

### Procedural characteristics

Throughout the study, a total of 163 procedures were performed on 123 patients underwent. The mean number of procedures was 1.3 per patient; most index procedures were PVI only (75%), with 25% of patients undergoing additional lesions. Further procedural details are given in Table 4.

**Table 4:** Procedural characteristics.

Procedure count (*n* = 163)	Patients (%)
1	93 (75.6)
2	22 (17.9)
3	6 (4.9)
4	2 (1.6)
No. of procedures per patient, mean ± SD	1.3 ± 0.6
Index procedure	
PVI only	93 (75.6)
PVI “plus”	30 (24.4)
CTI	14
Roofline	10
Posterior wall isolation	4
Carina	3
CFAE	1
Slow pathway modification	1
MIA anterior	2
FAT	2
RF time (mean ± SD)	29.3 ± 6.1
Fluoroscopy time (mean ± SD)	6.7 ± 4.6
Fluoroscopy dose (mean ± SD)	31.1 ± 34.3
Fluoroscopy DAP (mean ± SD)	281.9 ± 308.6

PVI plus, additional ablation lesions beyond the isolation of the pulmonary veins; PW, posterior wall box isolation; CFAE, complex fractionated atrial electrograms; MIA, mitral isthmus ablation; FAT, focal atrial tachycardia.

### Repeat procedures

**Figure 3:**
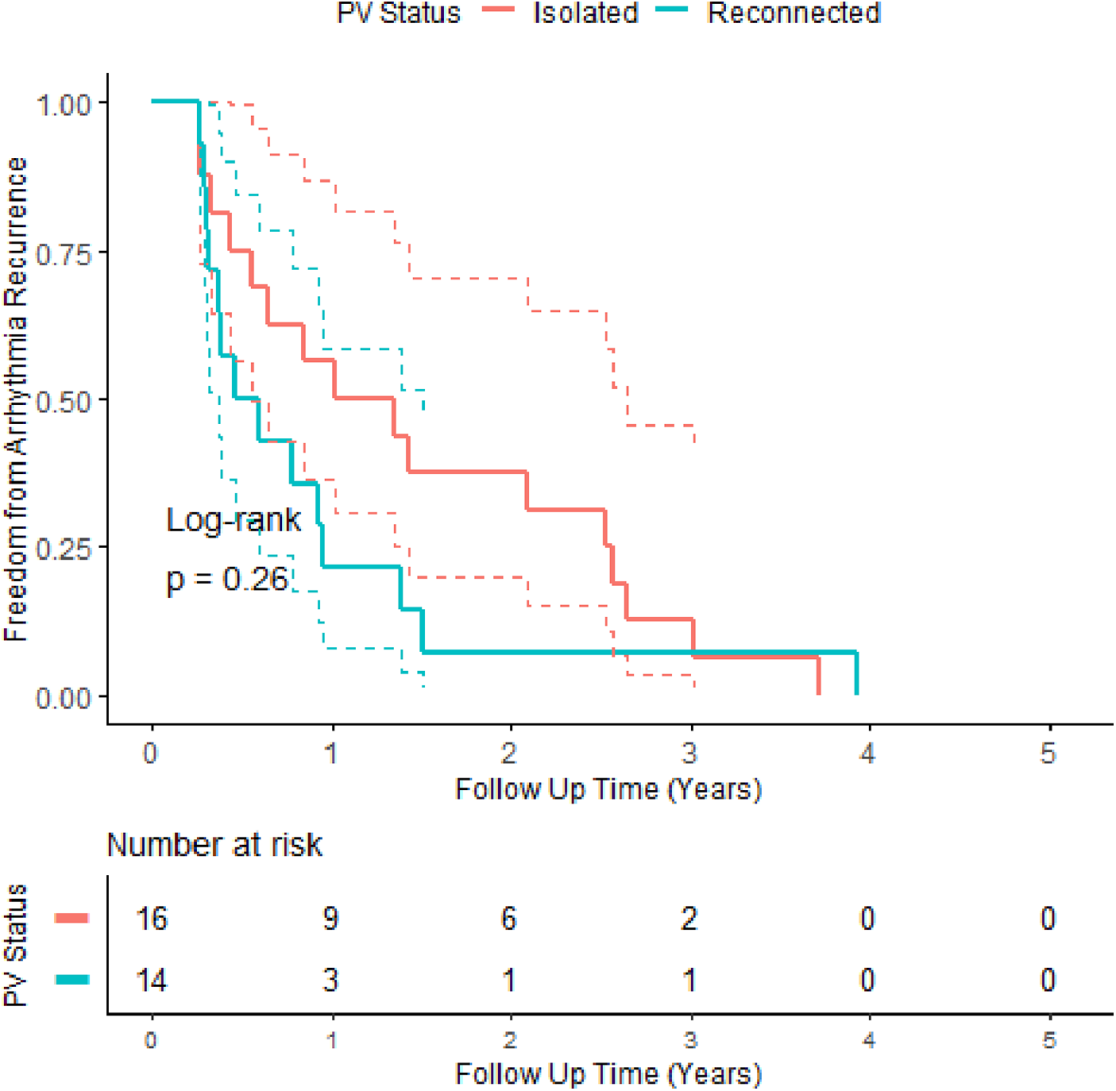
Kaplan–Meier survival curves of patients who underwent repeat procedures, up to their first recurrence only, stratified by the pulmonary vein connection status at the time of the first repeat procedure. Patients with connected veins tended to recur earlier, with the majority in the first year.

Of 123 patients, 30 (24%) underwent more than one procedure. Patients requiring multiple procedures had poorer outcomes (log-rank *p*-value: <0.0001), even allowing for multiple procedures. Remapping the pulmonary veins at the time of the first repeat procedure revealed that 16 patients (53.3%) had no reconnection, 10 (33.3%) had a right PV reconnection only, three (10%) patients had both veins reconnected, and only one (3.3%) patient had a reconnection in the left-sided veins only. Patients with reconnected veins at the time of their first repeat procedure had earlier recurrence (after the initial procedure) compared to patients with isolated veins, with most of these recurrences in the first year (11/14 patients)—see Figure 3. There was no significant difference in arrhythmia-free survival between patients with reconnected veins or not at the time of repeat procedure throughout the study (log-rank *p*-value: 0.12); however, in the first 2 years, patients with reconnected veins at repeat procedure showed a trend towards better outcomes with no further recurrence until into the second year. The mean number of extrapulmonary vein targets per patient undergoing repeat ablation was 1.53, the most common being posterior wall isolation in 17 patients, followed by anterior mitral isthmus line in 11 patients.

### Complications and procedural safety

Four patients had self-resolving vascular minor complications in the form of groin haematomas. Two patients experienced major (20) complications: one in the form of pericarditis requiring pericardiocentesis and steroid therapy, and a second patient with a right phrenic nerve injury. This resulted in a minor vascular complication rate of 4 per 154 procedures (2.5%) and a major complication rate of 2 per 154 procedures (1.25%).

### Predictors of recurrence

**Figure 2:**
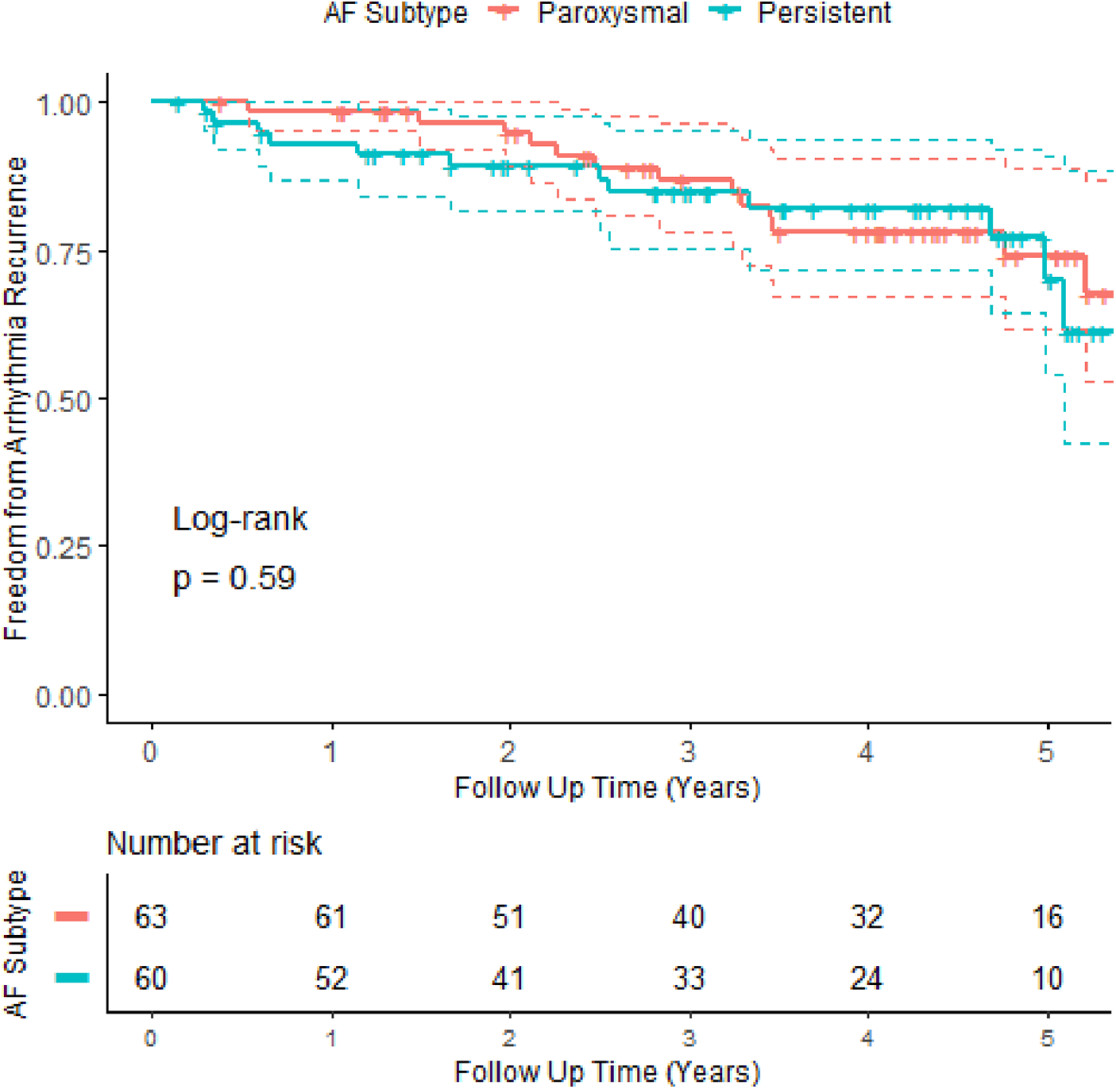
Kaplan–Meier survival curves with 95% confidence intervals for paroxysmal vs. persistent atrial fibrillation. An initial trend of better outcomes in paroxysmal AF does not continue beyond 2 years. Overall survival curves are not statistically different. +denotes censor.

**Figure 4:**
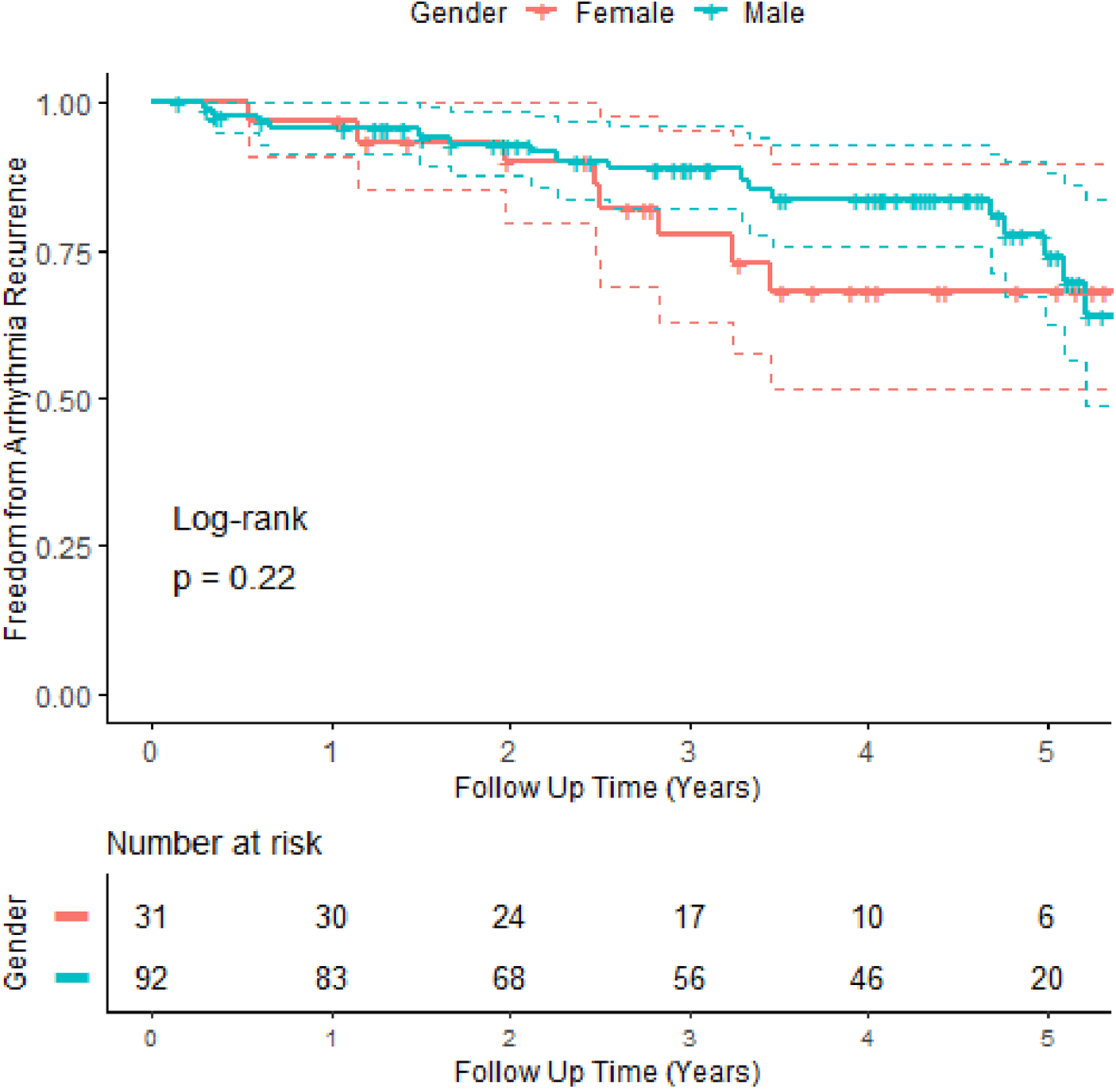
Kaplan–Meier curves comparing arrhythmia-free survival of male vs. female patients and allowing for repeat procedures.

Univariate and multivariate regression analyses (see Table 1) showed that age >75 years [HR: 2.7 (1.14–6.7)], BMI >35 kg/m^2^ [HR: 4.6 (1.8–11.4)], and an LA width above the third quartile in size [HR: 2.4 (1–5.7)] were independent statistically significant predictors of worse outcomes. Recurrence of arrhythmia in the blanking period (*p* = 0.1) showed a trend towards worse outcomes [HR: 1.9(0.89–4)], a finding that has been noted in previous work (21). There was no difference in recurrence rates between AF subtypes (Figure 2) or gender (Figure 4). Patients with reconnected veins at the time of repeat procedure tended to have earlier recurrence (Figure 3).

## Discussion

This study provides insights into the long-term efficacy and safety of ablation index-guided AF ablation for atrial fibrillation. Although long-term data are available on select patient cohorts (22), for the first time, this study presents long-term follow-up (beyond 5 years) data in a cohort of real-world consecutive patients utilising the ablation index and a pragmatic pre-defined ablation escalation strategy. Five-year data are particularly useful when counselling patients on treatment options as a common concern for them is the prognosis beyond the typically studied 1-year window.

Our patients were older compared with similar studies [PRAISE (7): 61 **± **8, CLOSE (23): 62 **± **10, Casella et al. (24): 60 **± **12]. The body mass index (BMI) (29.1 **± **4.7) was generally higher in our patients [CLOSE (23): 26.6 **± **4.3, Casella et al. (24): 25.4 **± **3.2]. The CHADSVASc (2.1 **± **1.5) score also tended to be higher compared with PRAISE (23) (a median score of 1) and similar to patients in the CLOSE (23) study, where 41% had a score >2. The LA anteroposterior diameter (AP) (46.6 **±** 7.4 mm) diameter tended to be longer (although measured on CT rather than by echocardiography) in PRAISE (43 **± **5 mm), CLOSE (40.4 ± 4.4 mm and 39.9 ± 3.9 mm), and Casella et al. (24) (42.3 **± **7.7 mm). In summary, our patients tended to be older, with higher BMI, greater CHADSVASc scores, and larger LA sizes compared to similar studies.

With only a mean of 1.3 procedures per patient, we see an arrhythmia-free survival rate of 95.8% at 1 year, holding to 72.3% at 5 years. These survival rates are modestly lower compared to other studies, likely due to our slightly more challenging patient characteristics.

We observed that older (age >75 years) and morbidly obese (BMI >35 kg/m^2^) patients remain challenging groups of patients to treat with catheter ablation for atrial fibrillation; in the latter group, this reinforces the importance of lifestyle modification as an integral part of the overall treatment strategy (25). As previously documented (21), there is a possible association between arrhythmia in the blanking period and worse outcomes.

The major complication rate of only 1.25%, with no procedural-related deaths or strokes observed during this study, was quite favourable when compared with large registry studies (26, 27) and comparable to rates seen in published RCTs (28). The authors attribute the low vascular complication rate to the consistent use of vascular ultrasound for all cases, as also evidenced in large retrospective studies (29).

Patients with paroxysmal AF are often considered to be at an earlier disease stage in comparison to those with persistent AF with lower recurrence rates. However, in this study, while patients with paroxysmal AF appeared to do better in the first year, this trend did not reach statistical significance (*p* = 0.59). The paroxysmal and persistent survival function curves (see Figure 2) overlap shortly after year 2 and follow a similar trajectory from that point onwards. The lack of prognostic difference conveyed by AF subtypes has been noted previously (21) but remains somewhat at odds with the conventional wisdom. To further explore this, we stratified patients by AF subtypes to look for differences in other variables. Except for parameters related to left atrial size (persistent patients tend to have larger atria), there were no statistically significant differences between the two groups. We speculated that the difference between the subtypes may disappear over time as the less severe paroxysmal patients “catch-up” with persistent patients. However, survival analysis stopped at 1 or 2 years showed no statistical differences between subtypes (Figure 2). It may be that persistent AF truly represents a more advanced disease state, but the data from this observational study do not support this nor the concept that paroxysmal patients are less likely to have a recurrence compared to persistent patients.

Next, we considered gender differences; significantly more men than women were enrolled in the study. Although the gender balance (74.8% male) does not differ from that found in similar studies of ablation index-guided PVI [PRAISE study: 75% male (23), CLOSE study (23): intervention 62% vs control 72% male, Casella et al. (24): 74% male]. Women in this study tended to be older, with a higher proportion over 75 years. Consequently, their CHADSVASc score tended to be higher, although there was no difference in non-age-related components of the score. Men were more likely to have persistent atrial fibrillation. Men tended to have smaller left atrial anterior–posterior dimensions, width, and area when indexed by the body surface area (BSA). Women tended to have more procedures and had a trend towards more PVI plus lesion sets. Despite these differences, there was no difference in the survival curves of men vs. women—see Figure 4.

Regarding patients who underwent repeat procedures, there is near equipoise between patients with no pulmonary vein reconnection (51%) and those with at least one at the time of the repeat procedure; among those with reconnection, only one patient out of 14 had reconnection limited to just the left-sided veins. This suggests that further attention needs to be paid to the isolation of the right-sided pulmonary veins as they are the site of greater recurrence than the left-sided veins, a result that was mirrored in the acute recurrence patterns seen in Hussein et al. (7). Second, patients who have non-isolated veins at their repeat procedure tended to recur earlier than those with isolated veins (Figure 3), most within the first year [median: 0.52, IQR: (0.36–1.51) years], while those with isolated pulmonary veins have recurrence at a later date [median: 1.1, IQR: (0.55–2.65) years]. This signal (early time to recurrence) allows us to potentially delineate patients with recurrence due to pulmonary vein reconnection. As such, it offers a potential target for prioritisation in resource-constrained systems of repeat procedures for patients as there is a trend towards better outcomes in these patients, particularly in the first 2 years. Patients with later recurrence likely represent the progression of the disease and the development of clinically significant extrapulmonary vein triggers, which to date have remained recalcitrant to treatment by catheter ablation.

### Limitations

This was a single-centre retrospective observational study. High/very high-power short-duration RF ablation technology was not available when the procedures were performed nor were the recent advances in pulsed field ablation technology. Regarding the cohort of patients undergoing repeat procedures, this was relatively small (30 patients), so any inferences from this data should be tempered with caution and used for hypothesis generation only. With only routine clinical follow-up, it is likely that the recurrence rates have also been underestimated.

## Conclusion

In conclusion, ablation index-guided pulmonary vein isolation is a safe and effective means of treating atrial fibrillation in symptomatic drug-refractory patients, as evidenced by a 72.3% arrhythmia-free survival at 5 years with repeat procedures. These data are novel in terms of duration of follow-up and is a useful addition to the body of knowledge particularly in terms of counselling patients before CA. Elderly and morbidly obese patients remain a challenging cohort of patients to treat effectively. Patients with arrhythmia recurrence at an earlier stage (between 3 and 12 months) are more likely to have non-isolated pulmonary veins. Such earlier recurrence offers a potential target to prioritise patients for repeat procedures as these patients show a trend towards better outcomes compared to patients with later recurrence, although further work is required to show this conclusively.

## Data Availability

The original contributions presented in the study are included in the article/**Supplementary Material**; further inquiries can be directed to the corresponding authors.

## References

[1] Hindricks G Potpara T Dagres N Arbelo E Bax JJ Blomström-Lundqvist C 2020 ESC guidelines for the diagnosis and management of atrial fibrillation developed in collaboration with the European Association for Cardio-Thoracic Surgery (EACTS). Eur Heart J. (2021) 42(5):373–498. 10.1093/eurheartj/ehaa612 32860505

[2] Calkins H Hindricks G Cappato R Kim YH Saad EB Aguinaga L 2017 HRS/EHRA/ECAS/APHRS/SOLAECE expert consensus statement on catheter and surgical ablation of atrial fibrillation. Heart Rhythm. (2017) 14(10):e275–444. 10.1016/j.hrthm.2017.05.012 28506916 PMC6019327

[3] Lemola K Hall B Cheung P Good E Han J Tamirisa K Mechanisms of recurrent atrial fibrillation after pulmonary vein isolation by segmental ostial ablation. Hear Rhythm. (2004) 1(2):197–202. 10.1016/j.hrthm.2004.03.071 15851153

[4] Providência R Marijon E Combes S Bouzeman A Jourda F Khoueiry Z Higher contact-force values associated with better mid-term outcome of paroxysmal atrial fibrillation ablation using the SmartTouch^TM^ catheter. EP Eur. (2015) 17(1):56–63. 10.1093/europace/euu218 25280910

[5] Kautzner J Neuzil P Lambert H Peichl P Petru J Cihak R EFFICAS II: optimization of catheter contact force improves outcome of pulmonary vein isolation for paroxysmal atrial fibrillation. EP Eur. (2015) 17(8):1229–35. 10.1093/europace/euv057 PMC4535556 26041872

[6] Taghji P El Haddad M Phlips T Wolf M Knecht S Vandekerckhove Y Evaluation of a strategy aiming to enclose the pulmonary veins with contiguous and optimized radiofrequency lesions in paroxysmal atrial fibrillation: a pilot study. JACC Clin Electrophysiol. (2018) 4(1):99–108. 10.1016/j.jacep.2017.06.023 29600792

[7] Hussein A Das M Riva S Morgan M Ronayne C Sahni A Use of ablation index-guided ablation results in high rates of durable pulmonary vein isolation and freedom from arrhythmia in persistent atrial fibrillation patients. Circ Arrhythm Electrophysiol. (2018) 11(9):e006576. 10.1161/CIRCEP.118.006576 30354288

[8] Chen S Schmidt B Bordignon S Urbanek L Tohoku S Bologna F Ablation index-guided 50 W ablation for pulmonary vein isolation in patients with atrial fibrillation: procedural data, lesion analysis, and initial results from the FAFA AI High Power Study. J Cardiovasc Electrophysiol. (2019) 30(12):2724–31. 10.1111/jce.14219 31588620

[9] Das M Loveday JJ Wynn GJ Gomes S Saeed Y Bonnett LJ Ablation index, a novel marker of ablation lesion quality: prediction of pulmonary vein reconnection at repeat electrophysiology study and regional differences in target values. Europace. (2017) 19(5):775–83. 10.1093/europace/euw105 27247002

[10] Solimene F Schillaci V Shopova G Urraro F Arestia A Iuliano A Safety and efficacy of atrial fibrillation ablation guided by ablation index module. J Interv Card Electrophysiol. (2019) 54(1):9–15. 10.1007/s10840-018-0420-5 30058055

[11] Teres C Soto-Iglesias D Penela D Jáuregui B Ordoñez A Chauca A Personalized paroxysmal atrial fibrillation ablation by tailoring ablation index to the left atrial wall thickness: the “Ablate by-LAW” single-centre study—a pilot study. Europace. (2022) 24(3):390–9. 10.1093/europace/euab216 34480548

[12] Falasconi G Penela D Soto-Iglesias D Francia P Teres C Saglietto A Personalized pulmonary vein antrum isolation guided by left atrial wall thickness for persistent atrial fibrillation. Europace. (2023) 25(5):1–15. 10.1093/europace/euad118 37125968 PMC10228614

[13] Sousa PA Barra S Saleiro C Khoueiry Z Adão L Primo J Ostial vs. wide area circumferential ablation guided by the ablation index in paroxysmal atrial fibrillation. Europace. (2023) 25(6):1–9. 10.1093/europace/euad160 37345859 PMC10286571

[14] *WEBSTER^™^ Decapolar Catheter | Biosense Webster*. Available online at: https://www.jnjmedtech.com/en-EMEA/product/webster-decapolar-catheter (accessed June 16, 2022).

[15] *CARTO® 3D Mapping Technology | 3D Mapping | Biosense Webster*. Available online at: https://www.jnjmedtech.com/en-US/product-family/3d-navigation?items_per_page=20 (accessed June 16, 2022).

[16] *THERMOCOOL SMARTTOUCH® SF Catheter | Biosense Webster*. Available at: https://www.jnjmedtech.com/en-EMEA/product/thermocool-smarttouch-sf-catheter (accessed June 16, 2022).

[17] Marrouche NF Dresing T Cole C Bash D Saad E Balaban K Circular mapping and ablation of the pulmonary vein for treatment of atrial fibrillation: impact of different catheter technologies. J Am Coll Cardiol. (2002) 40(3):464–74. 10.1016/S0735-1097(02)01972-1 12142112

[18] Ouyang F Ernst S Chun J Bänsch D Li Y Schaumann A Electrophysiological findings during ablation of persistent atrial fibrillation with electroanatomic mapping and double lasso catheter technique. Circulation. (2005) 112(20):3038–48. 10.1161/CIRCULATIONAHA.105.561183 16275866

[19] R Development Core Team. R: A language and environment for statistical computing. Vienna, Austria: R Foundation for Statistical Computing (2013). Available online at: https://www.r-project.org/ (accessed April 5, 2022).

[20] Guhl EN Siddoway D Adelstein E Bazaz R Mendenhall GS Nemec J Incidence and predictors of complications during cryoballoon pulmonary vein isolation for atrial fibrillation. J Am Heart Assoc. (2016) 5(7):1–7. 10.1161/jaha.116.003724 PMC5015404 27444510

[21] Herczeg S Keaney JJ Keelan E Howard C Walsh K Geller L Classification of left atrial diseased tissue burden determined by automated voltage analysis predicts outcomes after ablation for atrial fibrillation. Dis Markers. (2021) 2021:5511267. 10.1155/2021/5511267 34257744 PMC8245248

[22] La Fazia VM Pierucci N Mohanty S Gianni C Della Rocca DG Compagnucci P Catheter ablation approach and outcome in HIV+ patients with recurrent atrial fibrillation. J Cardiovasc Electrophysiol. (2023) 34(12):2527–34. 10.1111/jce.16076 37746923

[23] Phlips T Taghji P El Haddad M Wolf M Knecht S Vandekerckhove Y Improving procedural and one-year outcome after contact force-guided pulmonary vein isolation: the role of interlesion distance, ablation index, and contact force variability in the ‘CLOSE’-protocol. Europace. (2018) 20(FI3):f419–27. 10.1093/europace/eux376 29315411

[24] Casella M Dello Russo A Riva S Catto V Negro G Sicuso R An ablation index operator-independent approach to improve efficacy in atrial fibrillation ablation at 24-month follow-up: a single center experience. J Interv Card Electrophysiol. (2020) 57(2):241–9. 10.1007/s10840-019-00587-y 31313089

[25] Peigh G Wasserlauf J Vogel K Kaplan RM Pfenniger A Marks D Impact of pre-ablation weight loss on the success of catheter ablation for atrial fibrillation. J Cardiovasc Electrophysiol. (2021) 32(8):2097–104. 10.1111/jce.15141 34191371 PMC9305992

[26] Cappato R Ali H. Surveys and registries on catheter ablation of atrial fibrillation: fifteen years of history. Circ Arrhythmia Electrophysiol. (2021) 14(1):108–17. 10.1161/CIRCEP.120.008073 33441001

[27] Steinbeck G Sinner MF Lutz M Müller-Nurasyid M Kääb S Reinecke H. Incidence of complications related to catheter ablation of atrial fibrillation and atrial flutter: a nationwide in-hospital analysis of administrative data for Germany in 2014. Eur Heart J. (2018) 39(45):4020–9. 10.1093/eurheartj/ehy452 30085086 PMC6269631

[28] Russo AM Zeitler EP Giczewska A Silverstein AP Al-Khalidi HR Cha YM Association between sex and treatment outcomes of atrial fibrillation ablation versus drug therapy: results from the CABANA trial. Circulation. (2021) 143:661–72. 10.1161/CIRCULATIONAHA.120.051558 33499668 PMC8032462

[29] Ding WY Khanra D Kozhuharov N Shaw M Luther V Ashrafi R Incidence of vascular complications for electrophysiology procedures in the ultrasound era: a single-centre experience over 10,000 procedures in the long term. J Interv Card Electrophysiol. (2022) 66(3):693–700. 10.1007/s10840-022-01386-8 36214805

