# Supplementary figures and images for: Long-term results of ablation index guided atrial fibrillation ablation: insights after 5+ years of follow-up from the MPH AF Ablation Registry
Source: Front Cardiovasc Med. 2024 Jan 16;10:1332868. doi: 10.3389/fcvm.2023.1332868 (PMC10825003; doi:10.3389/fcvm.2023.1332868)

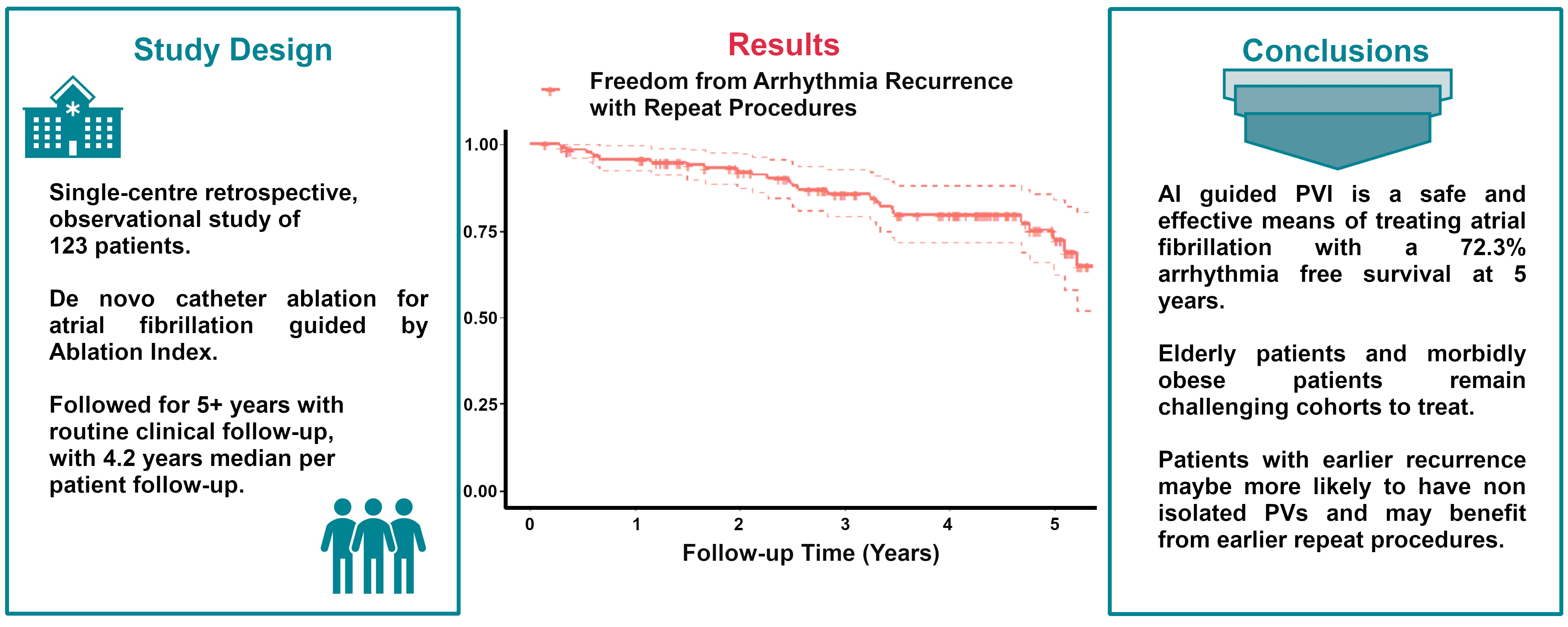

Supplement: Supplementary file 1 [file Image1.png]

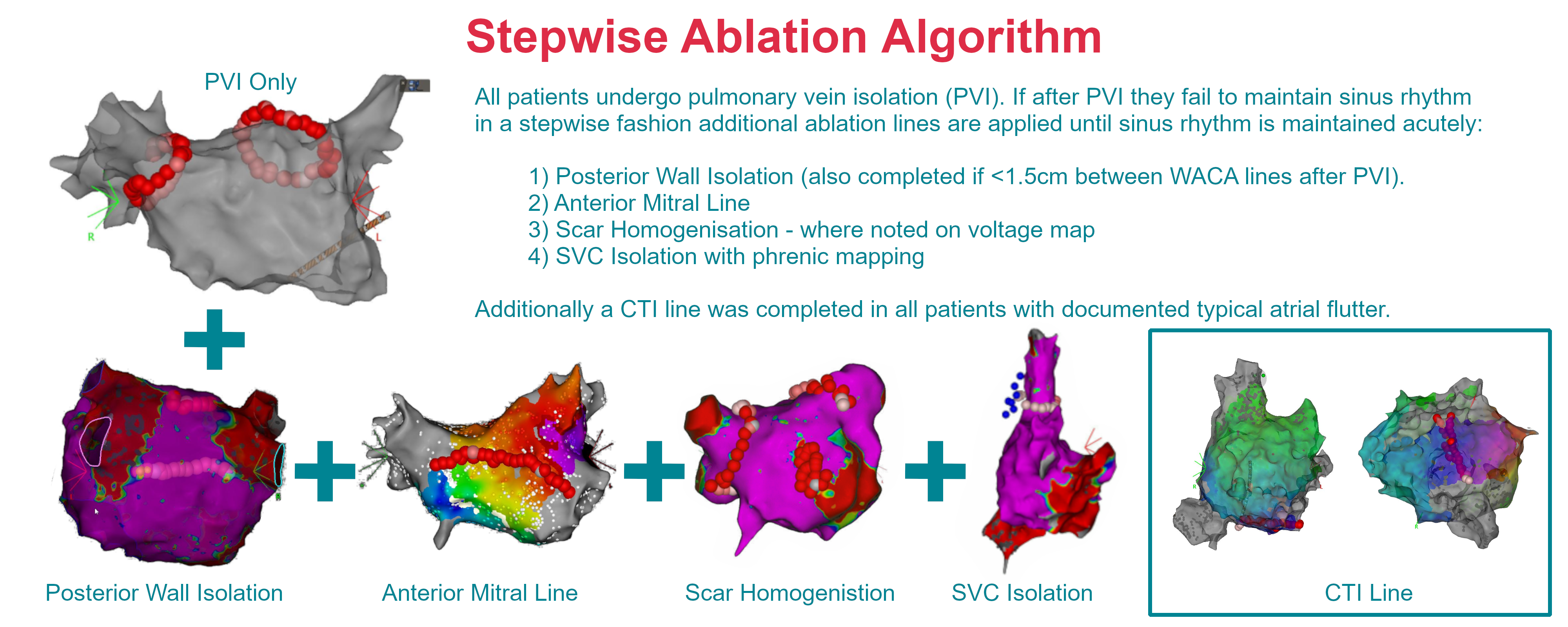

Supplement: Supplementary file 2 [file Image2.png]
